# Complete Genome Sequence of *Elizabethkingia* sp. BM10, a Symbiotic Bacterium of the Wood-Feeding Termite *Reticulitermes speratus* KMT1

**DOI:** 10.1128/genomeA.01181-15

**Published:** 2015-10-08

**Authors:** Dongmin Lee, Young-Kyoon Kim, Yeong-Suk Kim, Tae-Jong Kim

**Affiliations:** Department of Forest Products and Biotechnology, Kookmin University, Seoul, South Korea

## Abstract

*Elizabethkingia* sp. BM10 was isolated from the hindgut of the wood-feeding termite *Reticulitermes speratus* KMT1. It had cellobiohydrolase and β-glucosidase activities but not endo-β-glucanase activity. The complete sequence of its genome, which has a total size of 4,242,519 bases, is reported here. The genomic analysis identified six β-glucosidase candidate genes and three β-glucanase candidate genes.

## GENOME ANNOUNCEMENT

Wood-feeding termites are not only pests but are also natural models for efficient biological degradation of woods (1, 2). Their digestive systems have evolved with various symbiotic microbes, such as bacteria and protozoa (3, 4). Therefore, the functional contribution of symbiotic microbes is a critical part of the biological wood degradation in termites.

In a previous study (5), 16 bacterial species were isolated from the wood-feeding termite *Reticulitermes speratus* KMT1. Their endo-β-glucanase activity was barely detectable but their cellobiohydrolase and β-glucosidase activities were comparable to those of *Fomitopsis palustris* FFPRI 0507, a wood-decaying fungus (6). The lack of endo-β-glucanase activity may be a result of symbiotic adaptation in the hindgut of termites. Among these 16 species, *Elizabethkingia* sp. BM10 had strong cellobiohydrolase and β-glucosidase activities.

Here, we report the complete genome of *Elizabethkingia* sp. BM10, a symbiotic bacterium of the wood-feeding termite *R*. *speratus* KMT1. The genomic DNA of *Elizabethkingia* sp. BM10 was isolated using the cetyltrimethylammonium bromide method, and the genome was sequenced using a PacBio RS II system (Pacific Biosciences, Menio Park, CA, USA). The individual reads were assembled with the HGAP 2.0 (Pacific Biosciences). Prokka (7) was used to identify and annotate the genes.

The *Elizabethkingia* sp. BM10 genome was found to be composed of a chromosome that is 4,242,519 bp long and contains 3,893 coding sequences (CDSs), 55 tRNA genes, and 15 rRNA genes, with a GC content of 35.7%. The number of CDSs with a predicted function was 2,556 (65.66% of the total). The function analysis identified three candidate genes for endo-β-glucanase, that is, one gene (VO54_00280) for glycoside hydrolase family 5 and two genes (VO54_00275 and VO54_01019) for glycoside hydrolase family 16. Six candidate genes (VO54_00821, VO54_01398, VO54_03284, VO54_03419, VO54_03735, and VO54_03904) were identified for β-glucosidase. Among them, four genes are also found in the genome sequences of other *Elizabethkingia* strains, with more than 90% maximum identities. The other two genes, VO54_00821 and VO54_03735, were unique in *Elizabethkingia* sp. BM10, with maximum identities of 80% and 63%, respectively, in the NCBI database. The genome had two candidate enzyme genes (VO54_03451 and VO54_03884) for xylan degradation. It was found that, for nitrogen metabolism, *Elizabethkingia* sp. BM10 did not have a nitrogen fixation system but it had glutamine synthetase (VO54_01331) and glutamate synthase (VO54_00126, VO54_00127, and VO54_00236) for ammonia utilization.

Research on the genome of *Elizabethkingia* sp. BM10 will provide insights into symbiotic adaptation of the bacteria in the cellulose digestion of termites and the possible benefits of natural cellulose degradation.

### Nucleotide sequence accession number.

The complete genome sequence of *Elizabethkingia* sp. BM10 has been deposited in GenBank under the accession number CP011059.
